# Phytochemical and Insecticidal Activity of Some Thyme Plants’ Essential Oils Against *Cryptoblabes gnidiella* and *Scirtothrips mangiferae* on Mango Inflorescences

**DOI:** 10.3390/insects16090922

**Published:** 2025-09-02

**Authors:** Mohammad M. Aljameeli, Nawal Abdulaziz Alfuhaid, Ahmed Ramadan El-Rokh, Samira A. El-Salam, May A. Elhefni, Amira S. El-Rahman, Esraa M. Hussein, Jazem A. Mahyoub, Hayam Elshazly, Hanan S. Alyahya, Shatha I. Alqurashi, Mohamed A. Abdein, EL-Sayed M. Qaoud, Marwa M. Mosallam

**Affiliations:** 1 Department of Biological Sciences, College of Science, Northern Border University, Arar 73213, Saudi Arabia; 2 Department of Biology, College of Science and Humanities in Al-Kharj, Prince Sattam Bin Abdulaziz University, Al-Kharj 11942, Saudi Arabia; n.alfuhaid@psau.edu.sa; 3 Plant Protection Research Institute, Agricultural Research Center, Giza 12618, Egypt; ahmed.elrokh@arc.sci.eg (A.R.E.-R.); samira.adbelgalil83@arc.sci.eg (S.A.E.-S.); 4 Agricultural Chemistry Department, Faculty of Agriculture, Mansoura University, Mansoura 35516, Egypt; mayali@mans.edu.eg; 5 Horticulture Department, Faculty of Agriculture, Benha University, Moshtohor, Toukh 13736, Egypt; amira.abdelhamid@fagr.bu.edu.eg; 6 Horticulture Department, Faculty of Agriculture, Sohag University, Sohag 82524, Egypt; esraaelsyed@agr.sohag.edu.eg; 7 Department of Biological Sciences, Faculty of Science, King Abdulaziz University, Jeddah 21589, Saudi Arabia; jabdulrahman@kau.edu.sa (J.A.M.); halyahya@kau.edu.sa (H.S.A.); 8 Department of Biology, Faculty of Sciences & Arts-Scientific, Qassim University, Buraidah 52571, Saudi Arabia; hayam.elshazly@yahoo.com; 9 Department of Biological Sciences, Collage of Science, University of Jeddah, Jeddah 23218, Saudi Arabia; saaqurshi@uj.edu.sa; 10 Seeds Development Department, El-Nada Misr Scientific Research and Development Projects, Turrell, Mansoura 35511, Egypt; 11 Horticultural Department, Faculty of Agriculture, Suez Canal University, Ismailia 41522, Egypt; s_qaoud@agr.suez.edu.eg; 12 Department of Plant Production, Faculty of Environmental Agricultural Science, Arish University, Al Arish 45516, Egypt; marwa.mosalam@agri.aru.edu.eg

**Keywords:** thyme, mango inflorescences, insecticides, *Cryptoblabes gnidiella*, *Scirtothrips mangiferae*

## Abstract

Mango fruits are one of the strategic fruit crops in different countries that are attacked by several serious pests such as *Cryptoblabes gnidiella* and *Scirtothrips mangiferae*. Natural extracts, especially essential oils, provide several promising insecticide agents to control different insects as an alternative to synthetic insecticides. Using Clevenger-type hydrodistillation, the essential oils of five thyme plants—*Thymus vulgaris, Origanum vulgare, Thymus argenteus, Thymus citriodorus,* and *Origanum syriacum*—from Saudi Arabia and Egypt were extracted, and GC/MS analysis was performed. In addition, some chemical parameters of the five species were determined, such as chlorophyll a, chlorophyll b, *β*-carotene, total antioxidant capacity, total phenols, and total flavonoids. Two compounds, thymol and carvacrol, were identified in *T. vulgaris* and *O. vulgare* at ratios of 69.45 and 64.82%, respectively. These major compounds were isolated and identified using ^1^H NMR analysis. The insecticidal potentials of the five essential oils and their pure isolated compounds were evaluated on *C. gnidiella* and *S. mangiferae* on mango inflorescences. The results showed that *T. vulgaris* and *O. vulgare* oils were the most potent against *C. gnidiella* (LC_50_, 183.33 and 164.68 ppm, respectively) and *S. mangiferae* (18.93 and 16.93 ppm, respectively). Thymol and carvacrol had the highest effect on both insects. Furthermore, the effect of thymol and carvacrol at LC_50_ values on some biochemical parameters of *C. gnidiella* was determined. Therefore, thymol and carvacrol from *Thymus* species are promising compounds that could be used as insecticides against the harmful insects *C. gnidiella* and *S. mangiferae* on mango inflorescences.

## Simple Summary

Mango crops face significant threats from pests such as *Cryptoblabes gnidiella and Scirtothrips mangiferae*. This study investigated the potential of using essential oils from five thyme plants—*Thymus vulgaris*, *Origanum vulgare*, *Thymus argenteus*, *Thymus citriodorus*, and *Origanum syriacum*—as natural insecticides. Essential oils were extracted and analyzed using hydrodistillation, revealing high concentrations of thymol and carvacrol, especially in *T. vulgaris and O. vulgare* oils. The insecticidal effects of these oils and the compounds isolated from them were tested on the target pests. The results indicated that *T. vulgaris and O. vulgare* oils were the most effective, with low lethal concentrations (LC_50_ values) being potent against both pests. This study also measured biochemical changes in pest enzymes at these concentrations. Overall, thymol and carvacrol show promise as natural insecticides, offering an alternative to synthetic options for protecting agricultural crops.

## 1. Introduction

Around the world, medicinal plants play a significant role in both medicine and the economy. In particular, folk medicine has created a large database that can be used to search for new drugs. Several species in the Lamiaceae family hold unique medical and economic significance due to their diverse content [1]. Because of their pharmacological and biological qualities, *Thymus* species are widely used as therapeutic herbs. Thyme plant leaves and flowering parts are widely used in traditional medicine as a tonic and in herbal tea, in addition to being antibacterial, carminative, and antitussive [2].

*Thymus* is a particularly species-rich genus with a very broad geographic distribution due to its diversity and adaptability. The physical characteristics and metabolism of different thyme plants affect their chemical composition. *T. vulgaris, O. vulgare, T. argenteus, T. citriodorus,* and *O. syriacum* are the five plants of thyme that exhibit chemical variations that are typified by distinct plant oil compositions, typically in the absence of physical differences. Every plant usually contains two main types of oil; the *Thymus* genus is known for its phenols and terpenes. Some of the main ingredients are thymol, carvacrol, γ-cymene, γ-terpinene, 1,8-cineole, camphor, linalool, borneol, eugenol, and borneal [3,4,5,6,7].

Recently, researchers have been drawn to thyme plants due to their high production of certain secondary metabolites, including terpenoids, phenylpropanoids, and fatty acid derivatives. The usage of these secondary metabolites in the beauty and medical fields is growing [8,9,10,11]. These volatile compounds present in secondary metabolites include thymol, which has antibacterial, antifungal, and antiseptic properties, and carvacrol, recognized for its significance as a disinfectant and anti-infective. According to the authors of [12], additional compounds include limonene and *α*-terpineol, present in soaps and cosmetics; 1,8-cineole, used in medicine; and 1-borneol, which naturally repels insects. Certain species of thyme also contain essential oils that have insecticidal, allelopathic, antioxidant, and antibacterial qualities [4].

Mango (*Mangifera indica* L.), one of the major fruit crops in India and other countries, is known as the king of the fruits, known for its delicious taste and high nutritional value [13]. It is susceptible to attacks from several pests, such as the honeydew moth (HM), *Cryptoblabes gnidiella* Mill [14], and mango thrips, *Scirtothrips mangiferae* Priesner [15]. The Mediterranean region is home to the opportunistic *C. gnidiella* [16]. Worldwide, *C. gnidiella* has been observed to attack many hosts, including pomegranate, mangoes, grapes, avocado, citrus, and various vegetable crops [16,17]. Recently, Abdel Kareim et al. [14] observed high populations of the pest on mango inflorescences in Egypt, resulting in significant damage, especially when accompanied by mealybugs. According to Harari et al. [18], *C. gnidiella* can cause indirect damage to clusters by invading wounded berries with fungi, as well as direct damage when the larvae feed on the berries. This insect has caused losses in crop production of up to 30%. Mango thrips (*S. mangiferae*) have been known to attack mango in Egypt, Greece, Libya, Aden, Israel, and Sudan [15]. In addition to causing feeding damage, thrips play a significant role in the spread of viruses to plants, making them potentially dangerous pests [19]. Naturally occurring secondary metabolites have been promoted for use as insecticides due to growing concerns about the possibly harmful effects of artificial pesticides on humans and the environment. This study aimed to isolate the active principles from essential oils and evaluate their insecticidal activity against mango thrips (*S. mangiferae*) and honeydew moths (*C. gnidiella*) under laboratory conditions. Additionally, it assessed certain biochemical parameters, in light of renewed interest in finding natural insecticides or biopesticides derived from plants and the important role essential oils play in this field.

## 2. Materials and Methods

### 2.1. Chemicals and Instruments

Materials used: Aluminum sheets with TLC silica gel Merck GF254 precoated plates measuring 20 × 20 cm (Darmstadt, Germany). Petroleum ether (40–60 °C) (PE), ethyl acetate (EA), methylene chloride (MC), and methanol (MeOH) were acquired from LOBA CHEMIE PVT. Ltd. in Mumbai, India. Spray reagent for *P*-anisaldehyde. NMR analyses were performed in CDCl_3_ using a Bruker (400 MHz, AQ 4.089 s, Billerica, MA, USA). A UV–Visible spectrophotometer (model MA9523-SPEKOL 211, ISKRA, Horjul, Slovenia) was used for the spectrophotometric measurements.

### 2.2. Plant Materials

Egypt (*Thymus vulgaris* and *T. argenteus*) and the Kingdom of Saudi Arabia (*Origanum vulgare*, *T. citriodorus,* and *O. syricum*) provided the three *Thymus* species and two *Origanum* species seedlings. After two months of growth from seedlings, thyme plants were taken from the greenhouses and identified by the Biotechnology Research Lab, H.R.I., A.R.C., Giza, Egypt, and the Vegetable and Medicinal and Aromatic Plants Research Departments, Dokki, Giza, Egypt [20].

### 2.3. Essential Oil Isolation

A fresh weight of 1.0 kg from each plant was used to extract essential oil in 4 L of distilled water via hydrodistillation using a Clevenger-type apparatus for 8 h. The obtained oils were dehydrated over anhydrous sodium sulfate and stored at −4 °C in the refrigerator until analysis, following the method by Mostafa et al. [21].

### 2.4. GC-MS Method

Essential oil analyses were performed using an Agilent 7890A GC (Santa Clara, CA, USA) equipped with a 5975 Inert MS with a triple-axis detector and a DB-5 capillary column (30 m × 0.25 mm × 0.25 μm film thickness). The GC oven temperature was programmed to maintain a temperature of 50 °C for 5 min before increasing to 300 °C at a rate of 7 °C/min. Injector and detector temperatures were 250 °C and 230 °C, respectively. The MS was set at 70 eV ionization energy with a mass–electron (*m*/*z*) range of 50–550. The splitless mode was used to inject the sample solution, and helium was used as a carrier gas at a flow rate of 1 mL/min. The mass spectra of compounds were interpreted using spectral matches from the NIST Mass Spectral Library (Standard Reference Data, National Institute of Standards and Technology, Gaithersburg, MD, USA) and Wiley Registry of Mass Spectral Data (John Wiley & Sons, Hoboken, NJ, USA).

### 2.5. Isolation and Characterization of Active Ingredient

The essential oils of *T. vulgaris* and *O. vulgare* (0.2 g) were isolated over silica gel column chromatography using hexane as mobile phase with increasing polarity by ethyl acetate to give three subfractions in both plants Subfraction II (110 and 90 mg) in both plants, respectively, were purified with preparative thin-layer chromatography using aluminum sheet silica gel Merck GF_254_ precoated plates (20 × 20 cm) via an eluent system with 100% methylene chloride to yield compounds (**37**) and (**38**) (R*f* 0.76, 21 mg, 13 mg), respectively.

Compound (**37**) was isolated from *T. vulgaris* as a pale-yellow oily residue (21 mg; R*f* 0.76), which gave a purple color upon reacting with an anisaldehyde spray reagent. GC/MS, *m*/*z* (rel. int.): 150 (30%) [M, C_10_H_14_O]^+^, 135 (100) [M-CH_3_]^+^, 115 (18) [M-3CH_3_]^+^, 107 (10) [C_7_H_7_O]^+^, 91 (20) [C_7_H_7_]^+^. ^1^H NMR (400 MHz, CDCl_3_, *δ*_H_, ppm, J, Hz): 6.76 (d, *J* 7.7 Hz, H-3), 7.11 (dd, *J* 7.7, 2.2 Hz, H-4), 6.54 (d, *J* 2.2 Hz, H-6), 3.20 (pent, *J* 6.8 Hz, H-7), 1.25 (d, *J* 6.8 Hz, 6H-8,9), and 2.26 (s, 3H-10). Compound (**38**) was isolated from *O. vulgare* as a pale-yellow oily residue (13 mg; R*f* 0.76), which gave a purple color upon reacting with an anisaldehyde spray reagent. GC/MS, *m*/*z* (rel. int.): 150 (37%) [M, C_10_H_14_O]^+^, 135 (100) [M-CH_3_]^+^, 115 (3) [M-3CH_3_]^+^, 107 (15) [C_7_H_7_O]^+^, 91 (20) [C_7_H_7_]^+^. ^1^H NMR (400 MHz, CDCl_3_, *δ*_H_, ppm, J, Hz): 6.66 (d, *J* 1.7 Hz, H-2), 6.72 (dd, *J* 7.7, 1.7 Hz, H-4), 7.03 (d, *J* 7.7 Hz, H-5), 2.82 (pent, *J* 6.9 Hz, H-7), 1.22 (d, *J* 6.9Hz, 6H-8,9), and 2.21 (s, 3H-10).

### 2.6. Assay of Some Chemical Composition Parameters

Six phytochemical parameters were assessed for the five thyme plants.

#### 2.6.1. Chlorophyll (a and b) and β-Carotene Determination

The method developed by Nagata and Yamashita [22] was used to measure the contents of *β*-carotene and chlorophylls a and b (ChlA, ChlB). Approximately 10 mL of 80% acetone was used to grind 0.2 g of each thyme leaf sample, which was then filtered using Whatman No. 1 filter papers. Using a UV spectrophotometer, the filtered extract was placed in a cuvette, and absorbance was measured at 663, 645, 505, and 453 nm. The contents of *β*-carotene, chlorophyll a, and chlorophyll b were calculated using the following formulas:

- The value of chlorophyll a is 0.999 A_663_ − 0.0989 A_645_
- *β*-Carotene = (0.216 A_663_ − 1.22 A_645_) − (0.304 A_505_ + 0.452 A_453_)
- Chlorophyll b = −0.328 A_663_ + 1.77 A_645_.

#### 2.6.2. Total Antioxidant Capacity, Total Phenol, and Total Flavonoid Determinations

Approximately 2 g of green leaves were ground in a mortar with 20 mL of 80% methanol to create a methanolic extract before filtering. The total antioxidant potential of extracts from thyme leaves was measured using Prieto et al.’s methodology [23]. In an Eppendorf tube, 1 mL of reagent solution (0.6 M sulfuric acid, 28 mM sodium phosphate, and 4 mM ammonium molybdate) was mixed with 0.1 mL of sample solution. After being sealed, the tubes were incubated for 90 min at 95 °C in a thermal block. The absorbance of each sample’s aqueous solution was measured at 695 nm in comparison to a blank once the samples had cooled to room temperature. Antioxidant capacities were expressed as equivalents of α-tocopherol and ascorbic acid, respectively.

The total phenolic content was measured by using a 100 mL volumetric flask containing 60 mL of distilled water, which was mixed with 1 mL of the sample, blank, or standard (gallic acid) in water. Mix in the 5.0 mL of FC reagent. Add 15 mL of 20% sodium carbonate solution after 1 min. Adjust the volume to 100.0 mL. After about 2 h at about 23 °C at 760 nm, read the color produced [24].

Twenty milliliters of methanol, 1 mL of 5% AlCl_3_ (wt/vol), and 2 mL of each sample or reference solution (quercetin in the range of 4.0–12.0 µg/mL) were added. The volume was then increased to 50 mL using methanol at 20 °C. At 425 nm, the absorbances were measured after 30 min according to Woisky and Salatino [25].

### 2.7. Rearing of Tested Insects

Samples of mango inflorescences were obtained from Kafr Al Baramoun Research Farm, Horticultural Research Institute, Mansoura, Dakahlia, Egypt. We ensured that the samples were free of any pesticides. Small mango seedlings were used to rear mango thrips, *S. mangiferae,* which were kept in cages (1.0 × 1.0 × 1.0 m) in a glass greenhouse until treatment began. Afterward, the Plant Protection Research Institute’s Taxonomy Department verified the identification of the insects.

For the honeydew moths, *C. gnidiella* after their emergence, pairs of females and males were placed in plastic boxes (25 × 25 × 10 cm) containing a piece of moistened cotton wool with a 10% honey solution and a piece of inflorescence for oviposition. The eggs were extracted daily using a sensitive hairbrush. When the eggs developed to the larval stage, the resulting larvae were daily provided with fresh flowers of mango until they reached to the tested larval stage [14].

### 2.8. Bioassay Activity

The spray method was used to assess the toxicity of the essential oils and isolated compounds on mango thrips and honeydew moths under laboratory conditions, based on the method by Ragab et al. [26] and Allam et al. [27] with some modifications. Five serial concentrations were prepared for each treatment using Tween 80 and distilled water (3 replicates/concentration). For thrips, ten healthy thrips nymphs were placed on a mango leaf disk (3 cm in diameter) in a Petri dish for each replicate. One milliliter of the test solution was sprayed onto each treatment. Ten *C. gnidiella* 2nd instar larvae were deposited in a Petri dish containing an appropriate portion of fresh mango inflorescence and then sprayed with 2 milliliters of the test solution. Only Tween 80 and distilled water were used in the control treatment on both insects. All treatments were kept in an incubator at 25 ± 2 °C and 60 ± 5% RH during the experiment. Abbott stated that mortality was counted and corrected after 24 h for thrips and 72 h for honeydew moth [28]. The LC_50_ and LC_90_ values, along with their confidence intervals and the slope of the regression lines, were provided by Finney [29]. Additionally, the Sun equation was used to calculate the toxicity index [30].

Concentration in ppm = Weight in g/Volume in mL × 10^6^

### 2.9. Biochemical Investigation of C. gnidiella

Enzyme activity measurement in insects is a useful method for understanding a number of biological processes, including stress reactions and insecticide resistance. The active isolated thymol **37** and carvacrol **38** were used to measure enzyme activity. Thirty individuals of 2nd instar *C. gnidiella* were sprayed with two milliliters of the thymol solution at the LC_50_ value; distilled water was also used to spray the control treatment. These experiments were replicated three times for each compound. According to Ragab et al. [26,31], the live insects were weighed and frozen in an appropriate tube following a 72 h treatment. The insect enzymes Acid Phosphatase (ACP) [32], Alkaline Phosphatase (ALP) [33], Glutathione *S*-Transferase (GST) [34], and Acetyl Choline Esterase (AchE) [35] were estimated. All enzyme activities were measured at the Analysis Unit, Plant Protection Research Institute, Agriculture Research Center, Mansoura, Egypt, using a spectrophotometer.

### 2.10. Statistical Analysis

One-way analysis of variance (ANOVA) was conducted using CoStat software (version 6.400, 798 Lighthouse Ave, PMB 320, Monterey, CA, USA). Fishers’ least significant difference (LSD, 0.05) test was used to calculate significant differences between the means. The LC_50_ and LC_90_ values were ascertained via probit analysis.

## 3. Results and Discussion

### 3.1. Identification of Compounds (***37***) and (***38***)

The major compounds (**37**) and (**38**) were isolated as oily substances from the essential oil of *T. vulgaris* and *O. vulgare*, respectively.

The mass spectrum of the compounds (**37, 38**) showed the presence of M^+^ ion at *m*/*z* 150 (30%), corresponding to the molecular formula C_10_H_14_O. The spectrum also showed an ion base peak at *m*/*z* 135 (100%) due to the expulsion of a methyl group. Additionally, the fragment peak at *m*/*z* 91 (20%) resulted from the formation of the tropylium ion formula C_7_H_7_.

**Scheme 1:**
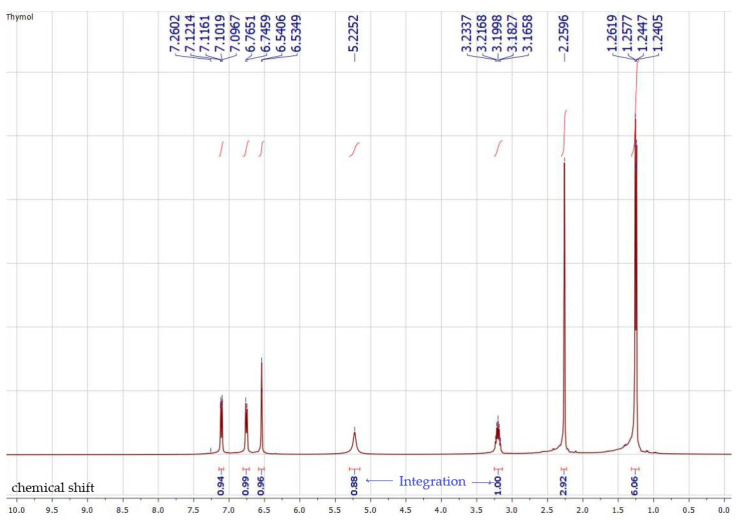
^1^H NMR of thymol **37** in CDCl_3_.

The ^1^H NMR data for compound (**37**) (Table 1, Scheme 1) displayed a proton signal pattern characteristic of a trisubstituted benzene ring moiety with an ABX system at *δ*_H_ 7.11 ppm (dd, *J* = 7.7, 2.2 Hz, H-4), 6.76 (d, *J* = 7.7 Hz, H-3) and at 6.54 (d, *J* = 1.8 Hz, H-6). In addition, the observation of an olefinic methyl proton signal at *δ*_H_ 2.26 (s, 3H-10) and an isopropyl unit at *δ*_H_ 3.20 (pent, *J =* 6.8 Hz, H-7), 1.25 (d, *J* = 6.8 Hz, 6H-8,9) confirmed the identification of compound (**37**) as thymol. A comparison of the spectral data of compound 37 with previously published data revealed that the structure was thymol.

**Scheme 2:**
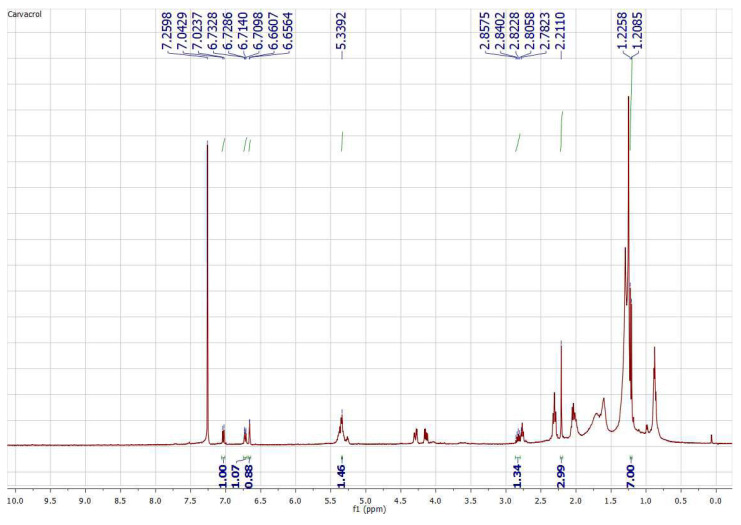
^1^H NMR of carvacrol **38** in CDCl_3_.

The ^1^H NMR spectrum (Table 1, Scheme 2) of compound (**38**) was similar to that of compound (**37**), but the olefinic methyl proton signal appeared at *δ*_H_ 2.21 (s, 3H-10) and the isopropyl unit at *δ*_H_ 2.82 (pent, *J* = 6.9 Hz, H-7), confirming the identification of compound (**38**) as carvacrol. This was in agreement with previously published spectral data from the literature [36,37].

### 3.2. GC/MS Analysis

The five essential oils extracted from the five plants of thyme—*T. vulgaris*, *O. vulgare*, *T. argenteus, T. citriodorus,* and *O. syriacum* were analyzed via GC/MS to identify their compounds. Table 2 displays the identification of 81 compounds from the chromatograms of these species by comparing their mass spectra with those of their analogs reported by the NIST library and Retention Index values (RI). These compounds can be classified mainly into three classes as follows: 37 monoterpenes including (**1**–**4**), (**6**–**12**), (**14**–**38**), and (**41**); 40 sesquiterpenes including (**39**–**40**), (**42**–**78**), and (**80**); two diterpenes (**79**) and (**81**); and two acetogenins (**5**) and (**13**). Two compounds—thymol (**37**) with ratios 69.45, 13.77, 36.87, 2.42, and 2.63%—and carvacrol (**38**)—with ratios 4.46, 64.82, 1.24, 4.54, and 13.00%—were found in high percentages in the five thyme plants. *p*-Cymene, eucalyptol, and linalool were found with percentages of 9.45, 8.53, and 7.10% in *T. vulgaris*, *O. vulgare*, and *T. argenteus*, respectively. γ-Eudesmol (**70**) and (-)-globulol (**75**) were also identified in *T. citriodorus* with high percentages of 10.02 and 23.06%, respectively. In addition to germacrene D (**53**), identified at 11.64%, and *δ*-cadinene (**59**), identified at 7.07%, they were also identified from *O. syriacum*. The essential oil of *T. vulgaris* mainly contains thymol, *p*-cymene, and carvacrol [38,39]. *O. vulgare* essential oil contains carvacrol and thymol as major compounds [40]. Another study on *T. vulgaris* oil from Saudi Arabia indicated the presence of thymol as a major compound (38.71%) when compared with *T. vulgaris* from Egypt (69.45%) [41]. Our study agreed with Walasek-Janusz et al. [42], indicating that carvacrol was a major compound in *O. vulgare* essential oil from four countries, albeit in different ratios. These findings are in line with previous research; however, the varying percentages may be attributed to morphological characteristics and metabolism, which influence the chemical composition of the samples.

### 3.3. Chemical Composition Assessment

The total chlorophyll (a and b), carotenoids, total phenolics, total flavonoids, and total antioxidant contents of the five thyme plants were assessed (Table 3). The ChlA, ChlB, and Carotene data indicated that *T. vulgaris* had significantly higher values at 0.969, 0.576, and 86.64 mg/100 g, respectively. Meanwhile, *T. argenteus* had significantly lower values of 0.182, 0.167, and 19.56 mg/100 g, respectively. On the other hand, the data on total phenol compounds varied significantly between thyme plants. The highest value was 0.677 mg/100 g for *O. syriacum* compared with the lowest values 0.151, 0.161, and 0.295 mg/100 g recorded for *T. citriodorus*, *T. vulgaris*, and *O. vulgare*, respectively. Flavonoid compounds varied significantly between thyme plants. The highest value was 1.034 mg/100 g for *O. vulgare* compared with other species. The total antioxidant content ranged between 43.36 and 23.86 mg/100 g. The highest value was recorded for *O. syriacum,* whereas the lowest was for *T. citriodorus*.

As shown in Table 3, all measured chemical parameters had high values in two plants, *T. vulgaris* and *O. vulgare*. These chemical parameters, including chlorophyll [43], carotenoids [44], and total flavonoids [45], are significant for environmental health, human well-being, and plant defense mechanisms. Chlorophyll and flavonoids play a crucial role in the biosynthesis of secondary metabolites, which enhance plant defense responses. Furthermore, the two active principles, thymol and carvacrol, have good insecticidal activity against the two insects under study. Thus, this information suggests that plants are safer when used as insecticides.

### 3.4. Insecticidal Activity

The toxicity of the five essential oils of thyme and two major compounds, thymol and carvacrol, was evaluated against two harmful insects (*S. mangiferae* and *C. gnidiella*). The results showed that the essential oils of *T. vulgaris and O. vulgare* were highly toxic against *S. mangiferae* (LC_50_, 18.93 and 16.93 ppm) and *C. gnidiella* (LC_50_, 183.32 and 164.68 ppm), respectively, compared with the other oils (Table 4 and Table 5). Furthermore, the isolated compounds, thymol and carvacrol, from *T. vulgaris* and *O. vulgare* were the most potent toward the two insects, respectively. Thyme is a plant whose essential oil is known for its insecticidal properties. Pavela et al. posited that the phenolic components of thyme essential oil are the main reason for its insecticidal activity [46]. According to Dargahi et al. [47]**,** Thyme plants exhibit potent insecticidal properties against a variety of insects, including *Aedes aegypti, Anopheles stephensi*, and *Staphilus oryzae*. The toxicity of both *T. vulgaris* and *O. vulgare* could be due to their chemical composition, particularly the presence of thymol (69.45%) and carvacrol (64.84%), respectively [47,48]. In addition, in a previous study, thymol and carvacrol from *T. vulgaris* had been evaluated for their strong insecticidal activity against *Alphitobius diaperinus* [49]. It is worth noting that these oils and their active ingredients are being used for the first time as insecticides on the insects under study.

### 3.5. Effect of Thymol and Carvacrol on C. gnidiella Enzyme Activity

The effect of thymol and carvacrol on the biochemical parameters (ACP, ALP, AchE, and GST) of *C. gnidiella* was determined (Table 6). The activity of acid and alkaline phosphatases decreased significantly in the presence of thymol and carvacrol compared with the control (Table 6). The hydrolytic enzymes (ACP and ALP) are responsible for separating phosphate groups from proteins and nucleotides under different conditions [50]. Additionally, the digestion and absorption efficiency of nutrients is affected by the activity of both ACP and ALP enzymes. The decrease in the ALP and ACP enzyme activity led to a reduction in the release of phosphate groups, thereby affecting metabolism and digestion functions [51]. AchE enzyme activity showed a significant decrease in the presence of thymol and carvacrol compared with the control (71.17, 83.50, and 316.1 µg AchBr/min/gm body weight, respectively). Maazoun et al. posited that phenolic compounds inhibited AchE activity and affected the insects’ nervous systems [52]. Furthermore, the detoxifying enzyme activity (GST) significantly decreased compared with the control. GST was considered the main detoxification enzyme responsible for insect resistance [53,54,55]. The inhibitory effect of both thymol and carvacrol on *C. gnidiella* AchE aligned with their effect on *Drosophila melanogaster* AchE [56]. The above results indicate that *C. gnidiella* is susceptible to both compounds (thymol and carvacrol), leading to insect death.

## 4. Conclusions

*Cryptoblabes gnidiella* and *Scirtothrips mangiferae* are serious pests that attack many strategic fruit crops such as mango trees. Using natural extracts as an alternative to synthetic insecticides to control these insects has become an urgent need. The essential oils of five thyme plants—*T. vulgaris, O. vulgare, T. argenteus, T. citriodorus,* and *O. syriacum*—were tested against *C. gnidiella* and *S. mangiferae*. Two compounds, thymol and carvacrol, were identified in all species at a high ratio, especially *T. vulgaris* and *O. vulgare* at 69.45 and 64.82%, respectively. Thymol and carvacrol were found to be the most toxic to both insects. Furthermore, a significant effect of the two compounds on certain biochemical parameters of *C. gnidiella*, including Acid Phosphatase, Alkaline Phosphatase, Glutathione S-Transferase, and Acetylcholine Esterase enzymes, was observed at LC_50_ values. Given its wide distribution and bioactive compounds like thymol and carvacrol, thyme shows strong potential as a botanical insecticide for crop protection.

## Figures and Tables

**Table 1:** ^1^H NMR of compounds **37** and **38** in CDCl_3_ (*δ*) [ppm] (Multiplicity, *J* [Hz]).

H-Atom	^1^H (Multiplicity, *J*) of Compound 37	^1^H (Multiplicity, *J*) of Compound 38
2	-	6.66 (d, *J* 1.7 Hz, 1H)
3	6.76 (d, *J* 7.7 Hz, 1H)	-
4	7.11 (dd, *J* 7.7, 2.2 Hz, 1H)	6.72 (dd, *J* 7.7, 1.7 Hz, 1H)
5	-	7.03 (d, *J* 7.7 Hz, 1H)
6	6.54 (d, *J* 2.2 Hz, 1H)	-
7	3.20 (pent, *J* 6.8 Hz, 1H)	2.82 (pent, *J* 6.9 Hz, 1H)
8, 9	1.25 (d, *J* 6.8 Hz, 6H)	1.22 (d, *J* 6.9 Hz, 6H)
10	2.26 (s, 3H)	2.21 (s, 3H)
-OH	5.23 (brs, 1H)	5.34 (brs, 1H)

**Table 2:** Chemical constituents of five thyme plants identified using the GC/MS technique.

Compound Name	RT	Mol. Formulae	Mol. Wt.	% of Compounds in Thyme Plants					
				1	2	3	4	5	RI
*α*-Thujene (**1**)	7.18	C_10_H_16_	136	0.10	-	0.07	-	-	910
*α*-Pinene (**2**)	7.36	C_10_H_16_	136	0.06	2.86	0.06	1.66	3.23	917
Camphene (**3**)	7.79	C_10_H_16_	136	0.07	0.29	0.07	0.06	0.34	932
*β*-Pinene (**4**)	8.65	C_10_H_16_	136	0.02	-	-	1.08	0.25	963
1-Octen-3-ol (**5**)	8.81	C_8_H_16_O	128	0.10	-	-	0.13	0.17	969
*β*-Myrcene (**6**)	9.11	C_10_H_16_	136	0.18	-	0.08	0.19	0.14	980
Yamogi alcohol (**7**)	9.51	C_10_H_18_O	154	-	-	-	2.02	0.39	995
*α*-Terpinene (**8**)	9.87	C_10_H_16_	136	0.09	-	-	0.13	0.82	1007
*p*-Cymene (**9**)	10.15	C_10_H_14_	134	9.45	-	1.85	1.06	1.16	1016
*D*-Limonene (**10**)	10.26	C_10_H_16_	136	0.13	-	0.68	0.44	-	1020
Eucalyptol (**11**)	10.33	C_10_H_18_O	154	0.24	8.53	0.77	-	1.88	1022
Santolina alcohol (**12**)	10.67	C_10_H_18_O	154	-	-	-	0.04	-	1033
1-Nonen-3-ol (**13**)	11.08	C_9_H_18_O	142	-	-	-	0.21	-	1046
*γ*-Terpinene (**14**)	11.20	C_10_H_16_	136	0.82	-	0.81		1.50	1050
trans-4-Thujanol (**15**)	11.48	C_10_H_18_O	154	0.41	1.06	0.56	-	-	1060
Linalool (**16**)	12.50	C_10_H_18_O	154	1.79	-	7.10	1.48	1.19	1092
Carveol (**17**)	13.19	C_10_H_16_O	152	-	-	0.48	-	-	1114
*α*-Campholenal (**18**)	13.34	C_10_H_16_O	152	-	-	-	0.16	-	1119
(*E*)-Pinocarveol (**19**)	13.73	C_10_H_16_O	152	-	-	0.15	1.48	0.03	1131
(+)-Camphor (**20**)	13.93	C_10_H_16_O	152	0.40	3.32	0.42	-	0.37	1136
Pinocarvone (**21**)	14.51	C_10_H_14_O	150	-	-	-	2.14	-	1156
(-)-Borneol (**22**)	14.58	C_10_H_18_O	154	1.44	1.18	1.24	-	0.23	1158
(*E*)-Pinanone (**23**)	14.83	C_10_H_16_O	152	-	-	-	-	0.06	1167
Terpene-4-ol (**24**)	14.93	C_10_H_18_O	154	0.52	-	0.46	0.42	0.35	1170
*p*-Mentha-1(7),8-dien-2-ol (**25**)	15.27	C_10_H_16_O	152	-	-	0.18	0.80	-	1181
*α*-Terpineol (**26**)	15.40	C_10_H_18_O	154	0.11	-	0.45	-	-	1185
(-)-Myrtenol (**27**)	15.54	C_10_H_16_O	152		-	-	0.83	0.11	1189
*cis*-Dihydrocarvone (**28**)	15.61	C_10_H_16_O	152	0.05	-	0.10	-	-	1192
Berbenone (**29**)	15.97	C_10_H_14_O	150	-	0.74	-	-	0.08	1204
Fenchyl acetate (**30**)	16.18	C_12_H_20_O_2_	196	-	-	0.26	0.13	0.03	1211
Thymol methyl ether (**31**)	16.61	C_11_H_16_O	164	1.31	-	0.67	0.80	0.06	1226
*p*-Cymene methyl ether (**32**)	16.88	C_11_H_16_O	164	1.01	-	3.70	-	0.22	1236
(-)-Carvone (**33**)	17.07	C_10_H_14_O	150	-	-	0.20	-	-	1242
*cis*-Geraniol (**34**)	17.30	C_10_H_18_O	154	-	-	0.28	0.47	-	1250
*β*-Citral (**35**)	17.79	C_10_H_16_O	152	-	-	-	0.18	-	1267
(-)-Bornyl acetate (**36**)	18.15	C_12_H_20_O_2_	196	-	0.82	1.32	0.21	0.21	1280
Thymol (**37**)	18.39	C_10_H_14_O	150	69.45	13.77	36.87	4.54	13.00	1288
Carvacrol (**38**)	18.68	C_10_H_14_O	150	4.46	64.82	1.24	2.42	2.63	1297
*α*-Cubebene (**39**)	19.92	C_15_H_24_	204	-	-	0.19	0.12	0.12	1342
*α*-Copaene (**40**)	20.67	C_15_H_24_	204	0.40	-	1.10	0.38	2.64	1368
Geranyl acetate (**41**)	20.86	C_12_H_20_O_2_	196	-	-	-	1.00	-	1375
*β*-Bourbonene (**42**)	20.93	C_15_H_24_	204	0.05	-	0.35	-	0.19	1377
*β*-Elemene (**43**)	21.11	C_15_H_24_	204	0.04	-	0.55	-	0.51	1383
*α*-Gurjunene (**44**)	21.62	C_15_H_24_	204	-	-	0.19	0.04	0.26	1401
Caryophyllene (**45**)	21.89	C_15_H_24_	204	2.47	0.82	2.56	2.19	5.10	1412
*trans*-*α*-Bergamotene (**46**)	22.26	C_15_H_24_	204	-	-	-	-	0.19	1426
Alloaromadendrene (**47**)	22.42	C_15_H_24_	204	-	-	-	0.02	0.03	1433
*α*-Muurolene (**48**)	22.55	C_15_H_24_	204	-	-	0.05	0.02	0.05	1437
Humulene (**49**)	22.80	C_15_H_24_	204	0.40	-	0.61	0.27	4.70	1447
*cis*-Muurola-4(15),5-diene (**50**)	23.03	C_15_H_24_	204	-	-	-	0.15	0.16	1457
Cadina-1(6),4-diene (**51**)	23.31	C_15_H_24_	204	-	-	0.07	-	0.24	1467
*γ*-Muurolene (**52**)	23.38	C_15_H_24_	204	0.10	-	0.11	0.13	0.38	1471
Germacrene D (**53**)	23.52	C_15_H_24_	204	0.88	-	4.25	2.16	11.64	1477
*α*-Selinene (**54**)	23.67	C_15_H_24_	204	0.06	-	-	-	0.85	1481
*γ*-Gurjunene (**55**)	23.93	C_15_H_24_	204	0.23	-	2.29	3.08	3.10	1492
*β*-Bisabolene (**56**)	24.17	C_15_H_24_	204	0.10	-	0.11	-	0.16	1501
*γ*-Cadinene (**57**)	24.37	C_15_H_24_	204	0.12	-	2.63	-	1.60	1509
Selina-3,7(11)-diene (**58**)	24.48	C_15_H_24_	204	-	-	-	1.46	-	1513
*δ*-Cadinene (**59**)	24.58	C_15_H_24_	204	0.60	-	1.38	-	7.07	1517
Kessane (**60**)	24.75	C_15_H_26_O	222	-	-	-	3.43	0.09	1523
Cubebol (**61**)	24.84	C_15_H_26_O	222	0.02	-	1.47	0.67	5.93	1527
*α*-Cadinene (**62**)	24.96	C_15_H_24_	204	-	-	-	-	0.20	1531
Caryophyllene oxide (**63**)	25.38	C_15_H_24_O	220	-	-	-	4.33	0.24	1556
Palustrol (**64**)	25.79	C_15_H_26_O	222	-	-	0.13	0.10	0.05	1563
Spatulenol (**65**)	26.02	C_15_H_24_O	220	0.09	-	0.45	2.47	0.48	1573
Isoaromadendrene epoxide (**66**)	26.15	C_15_H_24_O	220	0.42	-	0.53	2.01	0.49	1578
Salvial-4(14)-en-1-one (**67**)	26.42	C_15_H_24_O	220	-	-	0.12	-	-	1589
*β*-Oplopenone (**68**)	26.79	C_15_H_24_O	220	-	-	0.27	0.71	0.18	1604
*β*-Eudesmol (**69**)	27.07	C_15_H_26_O	222	0.06	-	0.08	-	-	1615
*γ*-Eudesmol (**70**)	27.31	C_15_H_26_O	222	-	-	-	10.02	0.95	1634
*T*-Cadinol (**71**)	27.55	C_15_H_26_O	222	0.42	-	0.65	-	3.47	1637
*δ*-Cedrol (**72**)	27.64	C_15_H_26_O	222	-	-	0.29	-	0.59	1641
*T*-Muurolol (**73**)	27.85	C_15_H_26_O	222	0.68	0.67	2.36	-	5.46	1650
Pogostole (**74**)	28.01	C_15_H_26_O	222	-	-	-	-	2.57	1656
(-)-Globulol (**75**)	28.26	C_15_H_26_O	222	-	-	0.73	23.06	0.25	1667
(*Z*)-*α*.-Atlantone (**76**)	29.31	C_15_H_22_O	218	-	-	1.70	0.11	0.40	1713
*α*-Cyperone (**77**)	30.14	C_15_H_22_O	218	-	-	-	0.60	0.29	1749
Hexahydrofarnesyl acetone (**78**)	31.92	C_18_H_36_O	268	-	-	-	0.84	2.43	1829
Isopimara-9(11),15-diene (**79**)	33.58	C_20_H_32_	272	-	-	1.37	0.35	0.39	1908
Aristol-1(10)-en-9-yl isovalerate (**80**)	33.92	C_17_H_36_O_2_	262	-	-	-	0.04	0.13	1925
Phytol (**81**)	37.35	C_20_H_40_O	278	-	-	-	0.50	-	2102
Total %				99.35	98.88	86.67	83.26	92.10	--

**R.T.**: Retention time; **Mol. Formulae**: Molecular formulae; **Mol. Wt.**: Molecular weight; **1**: *T. vulgaris*; **2**: *O. vulgare*; **3**: *T. argenteus*; **4**: *T. citriodorus*; **5**: *O. syriacum*; **RI**: Retention Index.

**Table 3:** Chemical composition of five thyme plants.

Thyme Plants	Chl A mg\100 g	Chl B mg\100 g	Carotene mg\100 g	Total Phenol mg\100 g	Flavonoids mg\100 g	Total Antioxidant mg\100 g
*T. vulgaris*	0.969 ^a^	0.576 ^a^	86.64 ^a^	0.161 ^b^	0.579 ^b^	28.61 ^c^
*O. vulgare*	0.890 ^b^	0.485 ^c^	83.26 ^b^	0.295 ^b^	1.034 ^a^	34.04 ^b^
*T. argenteus*	0.182 ^d^	0.167 ^e^	19.56 ^e^	0.516 ^a^	0.308 ^b^	26.47 ^d^
*T. citriodorus*	0.247 ^c^	0.230 ^d^	24.37 ^d^	0.151 ^b^	0.295 ^b^	23.86 ^e^
*O. syricum*	0.251 ^c^	0.535 ^b^	29.37 ^c^	0.677 ^a^	0.393 ^b^	43.36 ^a^
L.S.D. (0.05)	0.004	0.003	0.48	0.214	0.407	0.26

**a**, **b**, **c**, **d**, and **e**: Duncan’s letters. The same letter in the same column means non-significant.

**Table 4:** Toxicity of thyme essential oils and two major compounds, thymol and carvacrol, against mango thrips (*S. mangiferae*) after 24 h under laboratory conditions.

Treatment	LC_50_ (ppm)	Confidence Limit 95% (ppm)		LC_90_ (ppm)	Confidence Limit 95% (ppm)		Slope *± SE*	Toxicity Index (%) at LC_50_ Value
		Lower	Upper		Lower	Upper		
*T. vulgaris*	18.93	14.21	25.06	88.34	57.77	182.96	1.92 ± 0.30	40.94
*O. vulgare*	16.93	12.55	22.37	80.62	52.97	165.91	1.89 ± 0.29	45.78
*T. argenteus*	44.54	33.74	59.52	207.10	133.15	444.29	1.92 ± 0.30	17.40
*T. citriodorus*	89.60	66.20	119.54	448.01	287.94	969.56	1.83 ± 0.29	8.65
*O. syriacum*	77.38	57.92	102.98	371.16	239.93	789.53	1.88 ± 0.29	10.02
Thymol **37**	7.75	5.63	10.32	39.34	25.54	83.70	1.82 ± 0.29	100
Carvacrol **38**	8.45	6.36	11.04	37.03	24.98	71.66	2.00 ± 0.30	91.72

**Table 5:** Toxicity of thyme essential oils and two major compounds, thymol and carvacrol, against honeydew moth (*C. gnidiella*) after 3 days under laboratory conditions.

Treatment	LC_50_ (ppm)	Confidence Limit 95% (ppm)		LC_90_ (ppm)	Confidence Limit 95% (ppm)		Slope *± SE*	Toxicity Index (%) at LC_50_ Value
		Lower	Upper		Lower	Upper		
*T. vulgaris*	183.32	140.87	236.75	729.23	503.18	1339.02	2.14 ± 0.31	46.03
*O. vulgare*	164.68	121.89	217.35	779.77	514.61	1589.77	1.90 ± 0.30	51.24
*T. argenteus*	446.55	342.37	588.93	1913.89	1265.64	3847.49	2.03 ± 0.30	18.90
*T. citriodorus*	565.77	429.97	740.03	2439.76	1637.84	4757.78	2.02 ± 0.30	14.92
*O. syriacum*	479.07	367.52	619.88	1929.21	1325.72	3573.81	2.12 ± 0.31	17.62
Thymol **37**	87.46	66.23	113.94	373.74	253.31	714.83	2.03 ± 0.30	96.49
Carvacrol **38**	84.39	62.13	112.14	416.99	270.78	882.68	1.85 ± 0.29	100

**Table 6:** Enzyme activities of *C. gnidiella* larvae in the presence of thymol and carvacrol at the LC_50_ value.

Treatments	ACP (U/L) ± SE	ALP (U/L) ± SE	AchE (U/L/min) ± SE	GST (U/L) ± SE
Control	22.30 ± 1.13 ^a^	176.4 ± 3.49 ^a^	316.1 ± 14.09 ^a^	671.8 ± 22.34 ^a^
Thymol **37**	15.09 ± 0.56 ^b^	90.7 ± 5.41 ^b^	71.17 ± 6.21 ^b^	319.6 ± 9.68 ^c^
Carvacrol **38**	13.88 ± 0.75 ^b^	101.0 ± 3.11 ^b^	83.50 ± 0.29 ^b^	371.1 ± 2.54 ^b^
LSD, 0.05	2.94	14.29	30.78	48.92

**LSD**: least significant difference; **SE**: standard error; **a**, **b**, and **c**: Duncan’s letters. The same letter in the same column means non-significant.

## Data Availability

The original contributions presented in this study are included in the article. Further inquiries can be directed to the corresponding authors.

## References

[1] Amiri H. (2012). Essential Oils Composition and Antioxidant Properties of Three *Thymus* Species. Evid.-Based Complement. Altern. Med..

[2] Surburg H., Panten J. (2006). Common Fragrance and Flavor Materials.

[3] Borugă O., Jianu C., Mişcă C., Goleţ I., Gruia A., Horhat F. (2014). *Thymus vulgaris* essential oil: Chemical composition and antimicrobial activity. J. Med. Life.

[4] Ali I.B.E.H., Chaouachi M., Bahri R., Chaieb I., Boussaïd M., Harzallah-Skhiri F. (2015). Chemical composition and antioxidant, antibacterial, allelopathic and insecticidal activities of essential oil of *Thymus algeriensis* Boiss. et Reut. Ind. Crops Prod..

[5] Wu S., Wei F., Li H., Liu X., Zhang J., Liu J. (2013). Chemical composition of essential oil from *Thymus citriodorus* and its toxic effect on liver cancer cells. Zhong Yao Cai J. Chin. Med. Mater..

[6] Farhat M., Tóth J., Héthelyi B.É., Szarka S., Czigle S. (2012). Analysis of the Essential Oil Compounds of *Origanum syriacum* L. *Acta Fac*. Pharm. Univ. Comen..

[7] Badawy A.A., El-Mohandes M.A., Algharib A.M., Hatab B.E., Omer E.A. (2020). The essential oil and its main constituents of *Origanum syriacum* ssp. *sinaicum* grown wild in Saint Katherine Protectorate, South Sinai, Egypt. Al-Azhar J. Agric. Res..

[8] Thompson J.D., Stahl-Biskup E., Sáez F. (2002). Population structure and the spatial dynamics of genetic polymorphism in thyme. Thyme: The Genus Thymus.

[9] Boros B., Jakabová S., Dörnyei Á., Horváth G., Pluhár Z., Kilár F., Felinger A. (2010). Determination of polyphenolic compounds by liquid chromatography–mass spectrometry in *Thymus* species. J. Chromatogr. A.

[10] Sostaric I., Arsenijevic J., Acic S., Stevanovic Z.D. (2012). Essential Oil Polymorphism of *Thymus pannonicus* All. (Lamiaceae) in Serbia. J. Essent. Oil-Bear. Plants.

[11] Federici S., Galimberti A., Bartolucci F., Bruni I., De Mattia F., Cortis P., Labra M. (2013). DNA barcoding to analyse taxonomically complex groups in plants: The case of *Thymus* (Lamiaceae). Bot. J. Linn. Soc..

[12] Giron V., Garnatje T., Valles J., Perez-Collazos E., Catalan P., Valdes B. (2012). Geographical distribution of diploid and tetraploid cytotypes of *Thymus* sect. *Mastichina* (Lamiaceae) in the Iberian peninsula, genome size and evolutionary implications. Folia Geobot..

[13] Krishnamoorthy A., Visalakshi P.N.G. (2012). Record of Thrips on Mango. J. Hortic. Sci..

[14] Kareim A.I.A., Ragab M.E., Ghanim N.M., El-Salam S.A.A. (2018). Seasonal Activity, Natural Enemies and Life Table Parameters of *Cryptoblabes gnidiella* Mill. on Mango Inflorescences. J. Plant Prot. Pathol..

[15] Wysoki M., Ben-Dov Y., Swirski E., Izhar Y. (1993). The arthropod pests of mango in Israel. Acta Hortic..

[16] Dawidowicz Ł., Rozwałka R. (2016). Honeydew Moth *Cryptoblabes gnidiella* (Millière, 1867) (Lepidoptera: Pyralidae): An adventive species frequently imported with fruit to Poland. Pol. J. Entomol..

[17] Ben Yehuda S., Wysoki M., Rosen D. (1991). Phenology of the honeydew moth, *Cryptoblabes gnidiella* (Milliere) (Lepidoptera: Pyralidae), on avocado in Israel. Isr. J. Entomol..

[18] Harari A.R., Zahavi T., Gordon D., Anshelevich L., Harel M., Ovadia S., Dunkelblum E. (2007). Pest management programmes in vineyards using male mating disruption. Pest Manag. Sci..

[19] Grove T. (1999). Thrips Management in Mango Orchards. Ph.D. Thesis.

[20] Alqahtani M.M., Abdein M.A., El-Leel O.F.A. (2020). Morphological and Molecular Genetic Assessment of Some *Thymus* Species. Biosci. Biotechnol. Res. Asia.

[21] Mostafa M.E., Youssef N.M., Raghib H.M. (2023). Toxicity, Phytochemical Analysis and Biochemical Responses of Some Selected Plant Essential Oils Against Cotton Mealybug, *Phenacoccus solenopsis* Tinsley (Hemiptera: Pseudococcidae). Acad. J. Entomol..

[22] Nagata M., Yamashita I. (1992). Simple method for simultaneous determination of chlorophyll and carotenoids in tomato fruit. Nippon Shokuhin Kogyo Gakkaishi.

[23] Prieto P., Pineda M., Aguilar M. (1999). Spectrophotometric quantitation of antioxidant capacity through the formation of a phosphomolybdenum complex: Specific application to the determination of vitamin E. Anal. Biochem..

[24] Singleton V.L., Orthofer R., Lamuela-Raventós R.M. (1999). Analysis of Total Phenols and Other Oxidation Substrates and Antioxidants by Means of Folin-Ciocalteu Reagent. Methods Enzymol..

[25] Woisky R.G., Salatino A. (1998). Analysis of propolis: Some parameters and procedures for chemical quality control. J. Apic. Res..

[26] Ragab A., Taher M.A., El-Rafey H.H., El-Rokh A.R. (2024). Bioactive compounds from *Withania somnifera* dun and their toxicity against some piercing sucking pests. Appl. Biol. Chem..

[27] Allam R., Mohamed G.S., El-Solimany E., Ahmed E.E. (2023). Efficacy of some compounds against Thrips tabaci Lind. infesting onion plants at Sohag Governorate, Egypt. SVU-Int. J. Agric. Sci..

[28] Abbott W.S. (1925). A method of computing the effectiveness of an insecticide. J. Econ. Entomol..

[29] Finney D.J. (1971). Probit Analysis: A Statistical Treatment of the Sigmoid Response Curve.

[30] Sun Y.P. (1950). Toxicity index, an improved method of comparing the relative toxicity of insecticides. J. Econ. Entomol..

[31] El-Rokh A.R., Elhefni M.A., Elrafey H.H., Tadros L.K., Taher M.A. (2023). Phytochemical Profiling and Isolation of Bioactive Polyphenols from *Ipomoea carnea*. Egypt. J. Chem..

[32] Bakerman S. (1986). Textbook of Clinical Chemistry. N. W. Tietz, Ed., W. B. Saunders Co., Philadelphia, PA 19105, September 1985, xxvi + 1919 pp. Clin. Chem..

[33] Klein B., A Read P., Babson A.L. (1960). Rapid Method for the Quantitative Determination of Serum Alkaline Phosphatase. Clin. Chem..

[34] Habig W.H., Pabst M.J., Jakoby W.B. (1974). The First Enzymatic Step in Mercapturic Acid Formation. J. Biol. Chem..

[35] Simpson D.R., Bull D.L., Lindquist D.A. (1964). A Semimicro-technique for the Estimation of Cholinesterase Activity in Boll Weevils. Ann. Entomol. Soc. Am..

[36] Komaki Y., Tsukamoto T., Oishi Y., Shibasaki Y. (2022). Green polymer synthesis and low dielectric properties obtained by oxidative polymerization of thymol with CuCl-2-(p-tolyl)pyridine catalyst. React. Funct. Polym..

[37] Wang L.-L., Zhao H.-D., Lin H., Duan X.-Y., Xing G.-S., Xu W.-G., Qiao W., Zhao W.-J., Tang S.-A. (2020). Anti-inflammatory Constituents of *Dichapetalum longipetalum*. Chem. Nat. Compd..

[38] Porte A., Godoy R.L.O. (2008). Chemical composition of *Thymus vulgaris* L. (Thyme) essential oil from the Rio de Janeiro State, Brazil. J. Serbian Chem. Soc..

[39] Allahverdiyev A.M., Bagirova M., Yaman S., Koc R.C., Abamor E.S., Ates S.C., Baydar S.Y., Elcicek S., Oztel O.N. (2013). Chapter 17—Development of New Antiherpetic Drugs Based on Plant Compounds. Fighting Multidrug Resistance with Herbal Extracts, Essential Oils and Their Components.

[40] Han F., Ma G.-Q., Yang M., Yan L., Xiong W., Shu J.-C., Zhao Z.-D., Xu H.-L. (2017). Chemical composition and antioxidant activities of essential oils from different parts of the oregano. J. Zhejiang Univ. B.

[41] Al-Asmari A.K., Athar M.T., Al-Faraidy A.A., Almuhaiza M.S. (2017). Chemical composition of essential oil of *Thymus vulgaris* collected from Saudi Arabian market. Asian Pac. J. Trop. Biomed..

[42] Walasek-Janusz M., Grzegorczyk A., Malm A., Nurzyńska-Wierdak R., Zalewski D. (2024). Chemical Composition, and Antioxidant and Antimicrobial Activity of Oregano Essential Oil. Molecules.

[43] Martins T., Barros A.N., Rosa E., Antunes L. (2023). Enhancing Health Benefits through Chlorophylls and Chlorophyll-Rich Agro-Food: A Comprehensive Review. Molecules.

[44] Elvira-Torales L.I., García-Alonso J., Periago-Castón M.J. (2019). Nutritional Importance of Carotenoids and Their Effect on Liver Health: A Review. Antioxidants.

[45] Ullah A., Munir S., Badshah S.L., Khan N., Ghani L., Poulson B.G., Emwas A.-H., Jaremko M. (2020). Important Flavonoids and Their Role as a Therapeutic Agent. Molecules.

[46] Pavela R., Vrchotová N., Tříska J. (2009). Mosquitocidal activities of thyme oils (*Thymus vulgaris* L.) against *Culex quinquefasciatus* (Diptera: Culicidae). Parasitol. Res..

[47] Dargahi L., Razavi-Azarkhiavi K., Ramezani M., Abaee M.R., Behravan J. (2014). Insecticidal activity of the essential oil of *Thymus transcaspicus* against *Anopheles stephensi*. Asian Pac. J. Trop. Biomed..

[48] Miri R., Ramezani M., Javidnia K., Ahmadi L. (2002). Composition of the volatile oil of *Thymus transcaspicus* Klokov from Iran. Flavour Fragr. J..

[49] Szczepanik M., Zawitowska B., Szumny A. (2012). Insecticidal activities of *Thymus vulgaris* essential oil and its components (thymol and carvacrol) against larvae of lesser mealworm, *Alphitobius diaperinus* Panzer (Coleoptera: Tenebrionidae). Allelopath. J..

[50] Nation J.L. (2022). Insect Physiology and Biochemistry.

[51] Goharrostami M., Sendi J.J., Hosseini R., Mahmoodi N.O.A. (2022). Effect of thyme essential oil and its two components on toxicity and some physiological parameters in mulberry pyralid *Glyphodes pyloalis* Walker. Pestic. Biochem. Physiol..

[52] Maazoun A.M., Ben Hlel T., Hamdi S.H., Belhadj F., Ben Jemâa J.M., Marzouki M.N. (2017). Screening for insecticidal potential and acetylcholinesterase activity inhibition of *Urginea maritima* bulbs extract for the control of *Sitophilus oryzae* (L.). J. Asia-Pacific Èntomol..

[53] Hu Z.-D., Feng X., Lin Q.-S., Chen H.-Y., Li Z.-Y., Yin F., Liang P., Gao X.-W. (2014). Biochemical Mechanism of Chlorantraniliprole Resistance in the Diamondback Moth, *Plutella xylostella* Linnaeus. J. Integr. Agric..

[54] Al-Harbi N.A., Al Attar N.M., Hikal D.M., Mohamed S.E., Latef A.A.H.A., Ibrahim A.A., Abdein M.A. (2021). Evaluation of Insecticidal Effects of Plants Essential Oils Extracted from Basil, Black Seeds and Lavender against *Sitophilus oryzae*. Plants.

[55] Alhaithloul H.A.S., Alqahtani M.M., Ahmed M.A.I., Hesham A.E.-L., Aljameeli M.M.E., Al Mozini R.N., Gharsan F.N., Hussien S.M., El-Amier Y.A. (2023). Rosemary and neem methanolic extract: Antioxidant, cytotoxic, and larvicidal activities supported by chemical composition and molecular docking simulations. Front. Plant Sci..

[56] Askin H., Yildiz M., Ayar A. (2017). Effects of Thymol and Carvacrol on Acetylcholinesterase from Drosophila melanogaster. Acta Phys. Pol. A.

